# Neural Representations of Reward-Related Memories Shift across Development

**DOI:** 10.1523/JNEUROSCI.1325-25.2026

**Published:** 2026-02-17

**Authors:** Alexandra O. Cohen, Susan L. Benear, Camille V. Phaneuf-Hadd, Lila Davachi, Catherine A. Hartley

**Affiliations:** ^1^Department of Psychology, Emory University, Atlanta 30322, Georgia; ^2^Department of Psychology, New York University, New York, New York 10003; ^3^Department of Psychology, Harvard University, Cambridge, Massachusetts 02138; ^4^Department of Psychology, Columbia University, New York, New York 10027; ^5^Center for Neural Science, New York University, New York, New York 10003

**Keywords:** development, fMRI, memory, neural representations, reward, RSA

## Abstract

Rewards signal information in the environment that is valuable and thus useful to remember. Rewards benefit memory across development, but how reward-associated memories are represented in the brain has not been well characterized. Here we conducted pattern similarity analyses of fMRI data in male and female participants aged 8–25 to elucidate how neural representations in key memory-related brain areas are influenced by reward and how these relationships change across childhood and adolescence. We found that reward information was reflected in pattern similarity during encoding in the ventral temporal cortex and in changes in similarity from encoding to retrieval in anterior hippocampus (aHC). Strikingly, aHC reward-sensitive representations also varied with age such that adults’ memory benefitted from stability of hippocampal representations, whereas younger participants’ memory improvements were associated with greater drift in representations over time. Moreover, across all participants, reward-related univariate activation in the ventral tegmental area was associated with a greater tendency toward representational drift in aHC. Taken together, our findings demonstrate that reward modulates neural memory representations and that the representational patterns supporting reward-motivated memory shift with age.

## Significance Statement

Rewards benefit memory across development, but how these memories are represented in the brain has not been well characterized. Here we looked at multivariate patterns of brain activity in children, adolescents, and adults and found that the reward level (high vs low) assigned to pairs of pictures influenced participants’ neural patterns both during learning and when they retrieved the pairs from memory. Strikingly, in the hippocampus, adults’ memory for high-reward pairs benefitted from pattern stability over time, while children and adolescents’ high-reward memory benefits were associated with greater change in hippocampal patterns from encoding to retrieval. These results demonstrate that neural representations of reward-associated memories change with age.

## Introduction

Rewards in the environment signal valuable experiences and promote memory retention across development. Reward and motivation to obtain reward enhance subsequent memory in adults (Wittmann et al., 2005; Adcock et al., 2006; Wolosin et al., 2012; Patil et al., 2017) and in children and adolescents (Davidow et al., 2016; Ngo et al., 2019a). Our recent work demonstrated that although rewards similarly enhance memory performance across age, the underlying neural activation and functional connectivity supporting memory differ across development (Cohen et al., 2022a). Variation in distributed neural representations of encoded information also relates to memory performance (Ritchey et al., 2013; Tompary et al., 2016; Lohnas et al., 2018) and can characterize memory at a granular level that may not be captured by univariate methods (Lee et al., 2013). However, it is not clear how rewards influence memory representations across development.

Studies characterizing neural representations of visual memoranda often examine multivariate activation patterns within ventral temporal cortex (VTC), which includes category-selective (e.g., for faces) subregions (Khan et al., 2011). Visual stimuli that share encoding contexts often exhibit more similar VTC encoding activation patterns (Xue et al., 2010; Xiao et al., 2020), which may facilitate recall by promoting similarity in evoked mental states. When VTC activation patterns during retrieval more closely resemble patterns during encoding, recall is more likely to be successful in adults (Kuhl et al., 2011), with VTC encoding-retrieval similarity increasing into adolescence (Schlichting et al., 2022). However, it remains unclear how reward impacts VTC representations and memory across development.

Hippocampal activation patterns also reflect memory representations. Relational memory, or the binding of items in memory, is a canonical function of the hippocampus (Davachi et al., 2003), which putatively uses a sparse and distributed encoding scheme (McClelland et al., 1995; Norman and O’Reilly, 2003; Wixted et al., 2014). This “pattern separation” function may be late-developing (Ngo et al., 2018) due to protracted hippocampal development (Lavenex and Banta Lavenex, 2013), reflected in improved relational memory performance across childhood (Ngo et al., 2019b). Whether differentiation or similarity in hippocampal pattern representations is associated with better memory may shift with development (Varga et al., 2025). The hippocampus represents items that share an encoding context more similarly, including abstract “contexts,” like shared attentional state or semantic content (Brunec et al., 2020). Reward can also serve as an encoding context, with items from the same reward level exhibiting greater hippocampal pattern similarity across encoding (Wolosin et al., 2013) and between encoding and retrieval (Zeithamova et al., 2018). The anterior hippocampus (aHC), in particular, plays an important role in associative and motivated memory (Adcock et al., 2006; Murty et al., 2017), including in childhood (Cohen et al., 2022a). Burgeoning developmental work suggests that hippocampal pattern similarity at encoding can reflect shared contexts (Benear et al., 2022) and relate to associative memory performance (Kazemi et al., 2022), but there is also developmental change in aHC pattern similarity (Kazemi et al., 2022). These findings suggest that aHC memory representations may be impacted by reward and vary with age.

The ventral tegmental area (VTA) dopamine neurons project to the hippocampus and are proposed to underpin reward-motivated memory (Lisman and Grace, 2005; Luo et al., 2011). In animal models, coordination between reward-related VTA dopaminergic signaling and hippocampal activity enhances goal-directed behavior (Ding et al., 2025), and VTA activation can modulate neural representations in downstream regions (Iwashita, 2014). In humans, associative memory performance is predicted by VTA–aHC and VTA–VTC postencoding connectivity (Tompary et al., 2015; Murty et al., 2017). While VTA responses to reward exhibit marked developmental change (McCutcheon et al., 2012; Kim et al., 2016), there is limited work characterizing VTA contributions to memory across human development or how reward-related VTA encoding activity may modulate hippocampal memory representations.

The present study used representational similarity analysis to compare neural pattern similarity between high- and low-reward encoding and retrieval trials in children and adolescents, who might exhibit differential reward sensitivity. We examined how reward modulated neural similarity within and across encoding and retrieval in VTC and aHC. We also investigated relationships with behavioral memory performance and VTA encoding activation. We hypothesized that reinstatement of neural representations from encoding to retrieval would be associated with better memory, that hippocampal memory representations would reflect reward value and relate to reward-related VTA activation, and that the relationship between neural patterns and memory performance might exhibit age-related change.

## Materials and Methods

### Participants

Analyses included 89 participants ages 8–25 years (*M*_age_ = 16.16; SD_age_ = 4.67; 45 F, 44 M). A target sample size of 90 participants distributed evenly across age and sex was determined for our prior publication (Cohen et al., 2022a) based on previous research that used similar or smaller sample sizes to examine brain–behavior relations across comparable ages (Van Den Bos et al., 2015; Insel et al., 2019; Callaghan et al., 2021), as well as a multiple regression power calculation implemented in the “pwr” package in R. This calculation indicated that a sample size of 90 would yield ∼90% power to detect a medium effect (defined using Cohen's *f*^2^) of neural pattern similarity on memory. This effect size has been reported in prior studies assessing relations between neural measures and associative memory. The final sample used in analyses excluded data from: eight participants with excessive motion (participants without at least one complete set of encoding and retrieval runs due to runs with 15% or more timepoints censored with >0.9 mm relative translational motion), seven participants that chose not to complete or prematurely terminate the fMRI scan, and five participants with incomplete data due to fMRI scanner malfunction. Participants were recruited from the local New York City community and self-identified as African American/Black (12.2%), Asian (24.4%), Caucasian/White (38.9%), more than one race (23.3%), and Hispanic (15.6%). Participants were right-handed and provided self- or parental report indicating no history of head injury, diagnosed psychiatric illness, developmental disability, serious neurological or medical illness, sensory impairment (e.g., vision or hearing loss), use of medications that impact central nervous system function or peripheral physiological responses (e.g., beta-blockers), or major contraindication for MRI. Informed written consent was obtained from participants ages 18 and over, and assent was obtained from minor participants, consistent with approved research procedures by New York University's Institutional Review Board. Written consent on behalf of the child was obtained from parents or guardians of participants under age 18 prior to study participation. Participants received $75 in compensation and up to $21 in bonus money for participating in two sessions. This sample of participants was used in two earlier reports focusing on different research questions (Cohen et al., 2022a,b).

### Experimental design and statistical analyses

**Figure 1.**
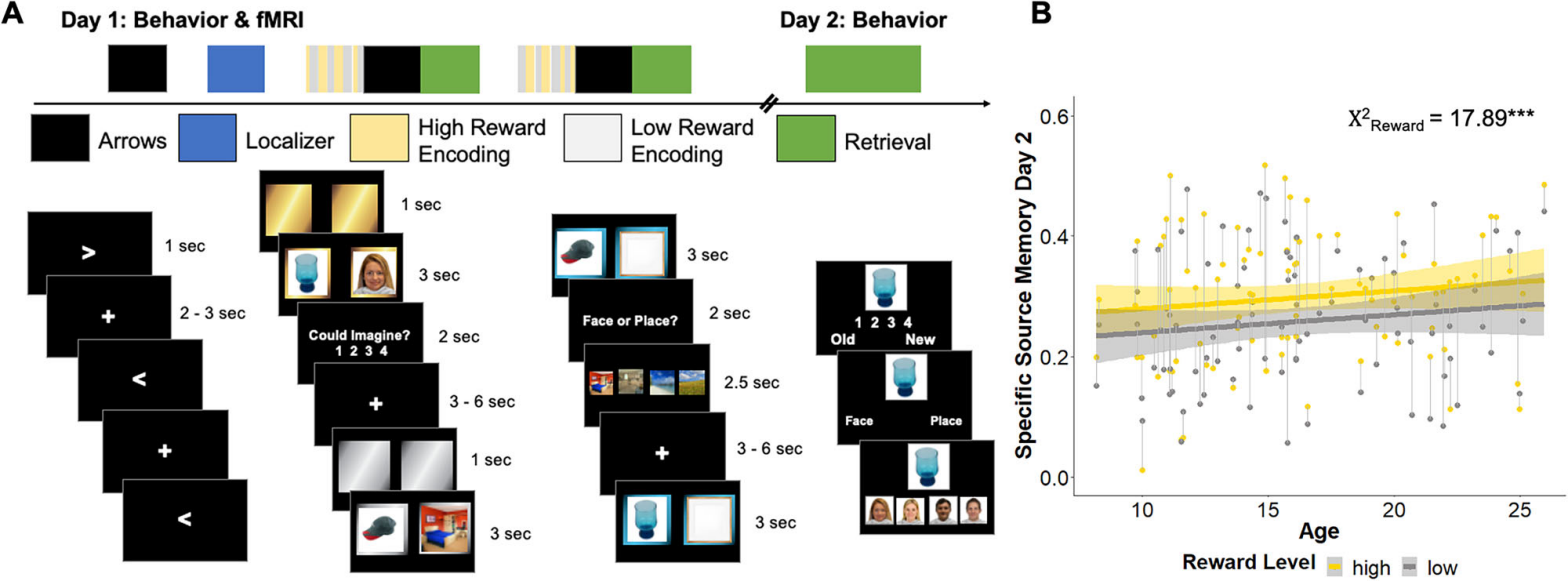
Experimental design. ***A***, Participants completed a high- and low-reward–motivated encoding and retrieval fMRI task. Participants also completed baseline and postencoding active rest (arrows) tasks as well as a face/place functional localizer task. After 24 h, they completed a behavioral retrieval task. ***B***, Participants showed better memory for high- relative to low-reward memoranda across age after 24 h. Shading depicts 95% confidence intervals around fitted lines. Thin gray lines connect individual subjects’ data points. ***p < 0.001.

Participants completed a reward-motivated encoding and retrieval task in the fMRI scanner (Fig. 1; Cohen et al., 2022a). All child and adolescent participants completed a mock scan to acclimate to the scanning environment prior to their fMRI scan. All participants completed a training that included instructions and sample trials for each scan. Tasks were programmed in Expyriment v0.9.1b2 (Krause and Lindemann, 2014) using Pygame v1.9.4 and Python v2.7.15. Images used in the reward-motivated encoding and retrieval task came from the RADIATE (Conley et al., 2018), Chicago Face Database (Ma et al., 2015), Harvard's Konkle Lab (Konkle et al., 2010), and MIT's Places Scene Recognition (Zhou et al., 2014) databases. After completing the training, participants underwent “pre-exposure” to the eight source images (four faces and four places) from the reward-motivated encoding and retrieval task to mitigate possible effects of source image category on memory performance (Mayes et al., 2007). Each source image was repeated five times each and for 3 s presentations (total duration, 3 min).

### Reward-motivated encoding and retrieval task

The reward-motivated encoding and retrieval fMRI task consisted of two runs of reward-motivated encoding and two runs of retrieval (Fig. 1*A*). Each encoding phase comprised 64 trials (32 high- and 32 low-reward) that each contained two images. To promote deep encoding, participants were instructed to tell themselves a story involving both images. On each trial, participants first saw two gold or silver squares for 1 s. The squares indicated whether remembering that the upcoming pair of images went together would help them win a big bonus of $15 (gold high-reward) or a small bonus of $1 (silver low-reward). A trial-unique picture of an object likely to be familiar to a child was overlaid on the left square, and one of the eight repeated source images was overlaid on the right square for 3 s. Source images were four faces (two women, two men) and four places (two outdoor scenes, two indoor scenes). The high-reward category of images (faces or places) was evenly distributed across age and sex. Following the stimulus presentation, participants had 2 s to rate how well they imagined their story on a scale from one (very easy to imagine) to four (very hard to imagine). A randomized, jittered ITI of 3–6 s (determined based on previous studies; Mumford et al., 2014) followed each 6 s trial. Each encoding run lasted 11.37 min.

After encoding and following a postencoding active rest scan (see Cohen et al., 2022a for active rest details), participants completed a retrieval scan. Each retrieval phase included half of the trial-unique objects from the preceding encoding block. Thus, each retrieval run consisted of 32 trials (16 high- and 16 low-reward from encoding) and lasted 6.57 min. On each trial, images were overlaid on blue squares that were perceptually similar to the gold and silver squares presented during encoding. An object from the preceding encoding phase was overlaid on the left square and a frame overlaid on the right square for 3 s. Participants were instructed to imagine the source image that had been paired with the object in the empty frame. Participants then had 2 s to report whether a face or a place belonged in the frame. Finally, they were shown the four source images from the selected category and selected the specific source image within 2.5 s. If a participant failed to respond, “Too slow!” was displayed on the screen for the remainder of the trial. As in encoding, each retrieval trial was followed by a random, jittered ITI of 3–6 s. Participants then completed a second set of encoding, active rest, and retrieval scans.

Participants also completed a behavioral memory retrieval test after 24 h (reported in Cohen et al., 2022a). The retrieval test consisted of all 128 objects from the motivated encoding task the day prior and 128 new objects. Half of the previously viewed images had been presented during both encoding and retrieval on Day 1, and the other half were only viewed once during encoding. On each trial, participants first saw one object and first indicated if it was definitely old, maybe old, maybe new, or definitely new. If the object was endorsed as definitely or maybe old, they then indicated whether the object had been paired with a face or place (hereafter, associative memory) and then which specific face or place (hereafter, specific source memory). If the object was endorsed as new, there were no further queries. The Day 2 retrieval test was self-paced.

### MRI data acquisition and preprocessing

MRI data were acquired at the NYU Center for Brain Imaging using a 3 Tesla Siemens Prisma scanner and a 64-channel head coil. High-resolution, T1-weighted anatomical scans were acquired using a magnetization-prepared rapidly acquired gradient echo sequence (TR, 2.3 s; TE, 2.3 ms; TI, 0.9 s; 8° flip angle; 0.9 mm isotropic voxels; field of view, 192 × 256 × 256 voxels; acceleration, GRAPPA 2 in the phase-encoding direction, with 24 reference lines) and T2-weighted anatomical scans using a 3D turbo spin echo sequence (T2, TR, 3.2 s; TE, 564 ms; echo train length, 314; 120° flip angle; 0.9 mm isotropic voxels; field of view, 240 × 256 × 256 voxels; acceleration, GRAPPA 2 × 2 with 32 reference lines in both the phase- and slice-encoding directions). Functional data were acquired with a T2*-weighted, multiecho EPI sequence (TR, 2 s; TEs, 12.2, 29.48, 46.76, 64.04 ms; MB factor, 2; acceleration, GRAPPA 2, with 24 reference lines; effective echo spacing, 0.245 ms; 44 axial slices; 75° flip angle; 3 mm isotropic voxels) from the University of Minnesota's Center for Magnetic Resonance Research (Feinberg et al., 2010; Moeller et al., 2010; Xu et al., 2013). Multiband with multiecho EPI sequences were used to aid with denoising of data and reducing signal dropout in subcortical brain regions. Total scan time was ∼1 h and 15 min, including short breaks between scans.

MRI data were preprocessed using fMRIPrep 20.0.6 (Esteban et al., 2019). The default options were used with slice timing disabled and MNI and T1w output spaces specified. The T1w space functional runs were used as input files in the reported analyses. Anatomical processing steps implemented via fMRIPrep included intensity nonuniformity correction, skull-stripping, spatial normalization, brain tissue segmentation, and surface reconstruction. FMRIPrep uses the tedana T2* workflow (Kundu et al., 2017; DuPre et al., 2021) to generate an optimally combined timeseries across echoes. This combined timeseries was then used in all subsequent preprocessing steps (e.g., registration estimation of head motion and confounds, susceptibility distortion estimation). All raw and preprocessed data were visually inspected. All subsequent processing and statistical analyses were completed in FSL version 5.0.10 (Jenkinson et al., 2012). Registration matrices were estimated by concatenating the transformations between the T1w functional to structural and structural to MNI space fMRIPrep outputs. Updated registration using these matrices derived from the fMRIPrep outputs was visually inspected.

### Encoding and retrieval fMRI analyses

Trial-specific activation patterns were modeled using the least squares single method (Mumford, 2013). Each generalized linear model (GLM) included a regressor for the trial of interest, a regressor with all other trials belonging to the same reward level as the trial of interest, a regressor with all trials belonging to the other reward level, and a regressor for onsets of no interest (during encoding, the onsets of the squares indicating the reward level and during retrieval, the onsets of the four possible source images). Each task regressor was convolved with a double gamma hemodynamic response function. In addition to timepoints censored for excessive motion, the following nuisance regressors derived from fMRIPrep for each run were also included in these models: average signal within anatomically derived eroded CSF mask, average signal within anatomically derived eroded white matter mask, six motion (translational and rotational) parameters and their derivatives, a framewise displacement regressor, the first six anatomical noise components (aCompCor), and the cosine components to perform high-pass filtering of the data. Data were spatially smoothed with a 3 mm FWHM Gaussian kernel. *T* statistic maps for each stimulus were then used in subsequent analyses.

Univariate encoding analyses used run-level GLMs for each participant. Each GLM had two task regressors: high-reward trials and low-reward trials. Each task regressor was convolved with a double gamma hemodynamic response function and included temporal derivatives. The following nuisance regressors derived from fMRIPrep for each encoding run were also included: six motion (translational and rotational) parameters and their derivatives, a framewise displacement regressor, the first six anatomical noise components (aCompCor), and the cosine components to perform high-pass filtering of the data. Encoding runs were combined using fixed-effect analyses. FSL's FLAME 1 was used to perform a group-level mixed–effect analysis. The group average was calculated for the high-reward > low-reward contrast of interest and included demeaned age and demeaned age squared as covariates.

### ROI definition

We investigated pattern similarity and encoding activation using several a priori ROIs that have been previously implicated in reward-motivated memory processes and in which there were significant effects in univariate or functional connectivity analyses from our prior paper using this dataset (Cohen et al., 2022a): VTC, aHC, and VTA. A VTC ROI was created using the Harvard-Oxford cortical probabilistic structural atlas available in FSL. The parahippocampal cortex, temporal fusiform cortex, and temporal occipital fusiform cortex were merged to create the VTC ROI. This ROI was then warped into subject T1w space and thresholded at 50%. Bilateral aHC was defined in standard space using a probabilistic atlas at conventional MRI resolution (Ritchey et al., 2015). The bilateral aHC ROI was warped into subject T1w space and thresholded at 75%. The VTA ROI was defined in standard space using a probabilistic atlas that reliably defines this area at conventional MRI resolution (Murty et al., 2014). The ROI was warped into subject T1w space and thresholded at 75%. All ROIs were visually inspected.

### Behavioral data analyses

Data processing and statistical analyses were conducted using R version 4.2.1 (de Micheaux et al., 2014). Mixed-effect models were run using the “lme4” package (Bates, 2011). Age was treated as a continuous variable in all analyses and was *z*-scored across all participants. We examined Day 2-specific source memory because we previously observed reward-motivated memory enhancements in this measure after the 24 h delay (Cohen et al., 2022a). Correct specific source memory was defined as trials where the specific source image (i.e., the specific woman, man, indoor place, or outdoor place) was accurately identified. Because Day 2-associative memory (i.e., face or place) was only queried on items identified as old, the denominator for the Day 2 measure was computed as the total number of items correctly identified as old for each participant. Trials were subdivided into paired associates that had been retrieved on both days or only on Day 2 to account for effects of testing on memory performance.

As reported previously (Cohen et al., 2022a), we fit models to the memory data using a mean-centered, scaled linear age predictor and a squared mean-centered, scaled age predictor to test for nonlinear effects of age. A likelihood ratio *χ*^2^ test showed that the data were not better fit by quadratic age models (*χ*^2^_(4)_ = 1.98; *p* = 0.74), indicating that a linear age model provided a better fit to the data. We fit a maximal model, including a single random intercept per participant and random slopes for within-subject fixed effects [reward level (high or low) and, for the Day 2 data, retrieval condition (retrieved Day 1 or not tested)] and their interaction, and simplified the models that failed to converge by removing random slopes until we identified the most complex random effects structure supported by the data (Bates et al., 2015). The model included a random intercept for participant and random slopes for retrieval condition. The high-reward category of source image (face or place) for each subject was included as a covariate of no interest in all analyses. Statistical significance of the fixed effects is reported from the analysis of deviance (Type III Wald *χ*^2^ tests using Satterthwaite approximations for degrees of freedom).

### Neural pattern similarity analyses

The input data for similarity analyses were *t* statistics extracted for each voxel of every trial within our ROIs of interest (VTC and aHC) that had been warped into subject T1w space. We computed three different pattern similarity measures within each ROI: encoding similarity, retrieval similarity, and encoding-retrieval similarity. Encoding similarity and retrieval similarity were computed as Pearson's correlations between every trial with every other trial within the same reward level (high or low) and memory phase (encoding or retrieval). This analysis was intended to capture the representation of a “brain state” associated with both the reward level and the memory phase. Importantly, these correlations were computed across runs to avoid overinflating correlation values. Thus, these analyses—and all subsequent analyses using these measures—included 73 subjects (16 subjects only had partial data; i.e., one of two encoding and/or retrieval runs). Encoding-retrieval similarity was computed as Pearson's correlations between corresponding encoding and retrieval activation patterns for each trial. Correlation scores were Fisher *r*-to-*z* transformed so that they could be submitted to further analyses and then averaged, resulting in measures of high-reward and low-reward pattern encoding similarity, retrieval similarity, and encoding-retrieval similarity.

Mixed-effect models examining whether pattern similarity in a priori ROIs varied as a function of the reward level and age were fit in the same manner as described for behavioral analyses above. All models converged with a random intercept per participant and were best fit by linear age models, except for the encoding-retrieval similarity model for VTC, in which the quadratic age provided a better fit to the data (*χ*^2^_(2)_ = 7.43; *p* = 0.024). However, due to only small differences in AIC (−635.77 vs −639.19), we followed up with a two-line test to investigate whether there was an inflection point in age, such that two lines with significant but different slopes were present on either side of this age value. We did not find evidence for such a “U-shaped” age effect (both slope *p*s > 0.09), suggesting only a subtle nonlinear age effect, but an overall increase in VTC ERS with age into early adulthood (Fig. S1). Statistical significance of fixed effects is reported from the analysis of deviance (Type III Wald *χ*^2^ tests using Satterthwaite approximations for degrees of freedom) performed on the *lmer* models.

### Brain–behavior relation analyses

Brain–behavior analyses comprised one trial-wise analysis using glmer and two multiple regression analyses using the lm function in base R (for model code, see Table S1). We examined the trial-wise relation between VTC encoding-retrieval similarity and memory after 24 h. This analysis was restricted to trials that were retrieved twice because we did not have neural retrieval data for items tested only once after 24 h. The glmer model comprised VTC encoding-retrieval similarity predicting memory accuracy and controlling for reward level, age, and the high-reward category of source image. This model converged with random slopes for the reward level and random intercepts for each participant. Statistical significance of fixed effects was reported from an analysis of deviance (Type III Wald *χ*^2^ tests using Satterthwaite approximations for degrees of freedom) performed on the *glmer* model. The first multiple regression analysis examined the relations between reward-sensitive neural similarity measures and the high-reward memory benefit (high-/low-reward–specific source memory after 24 h) in a single multiple regression including the following predictors: each of the two neural similarity measures (the absolute value of the difference score between high- vs low-reward aHC encoding-retrieval similarity and the difference score between high- vs low-reward VTC encoding similarity), age, and the high-reward category of source image. In a separate multiple regression, we then examined the relation between VTA encoding activation and high- versus low-reward aHC encoding-retrieval similarity, with VTA encoding activation predicting high- versus low-reward aHC encoding-retrieval similarity and modeling age as a covariate of interest, controlling for the high-reward category of source image.

## Results

### Reward-motivated memory enhancements are evident across age

We previously reported that this sample of participants showed better memory for specific high-reward relative to low-reward associations across all ages after 24 h (see Cohen et al., 2022a for a more thorough description of the memory results). Specifically, we ran a linear mixed-effect model examining specific source memory as a function of the reward level (high or low), age, and retrieval condition (whether the association was retrieved on both days or just Day 2), controlling for the high-reward source image category. There was a significant effect of the reward level (*χ*^2^_(1, *N* = 89)_ = 17.89; *p* < 0.001), with high-reward associations better remembered than low-reward associations. As expected, there was also a significant effect of retrieval condition, indicating better memory for associations that were retrieved twice rather than just once (*χ*^2^_(1, *N* = 89)_ = 49.56; *p* < 0.001). However, there was no reward level-by-retrieval condition interaction, so we collapsed across conditions for visualization and subsequent analyses (Fig. 1*B*). There were no other significant main effects or interactions (*χ*^2^s < 2.00; *p*s > 0.15). When we restricted this analysis to trials that were retrieved only during Day 1, we saw a similar pattern of results. There was a significant effect of the reward level (*χ*^2^_(1, *N* = 89)_ = 6.50; *p* = 0.011), and here we also saw a marginal effect of age (*χ*^2^_(1, *N* = 89)_ = 2.92; *p* = 0.087), such that memory improved with age. There were no other significant main effects or interactions (*χ*^2^s < 0.70; *p*s > 0.40). These findings show that reward similarly enhances specific associative memory across age.

### VTC encoding similarity increases with age and greater reward value

**Figure 2.**
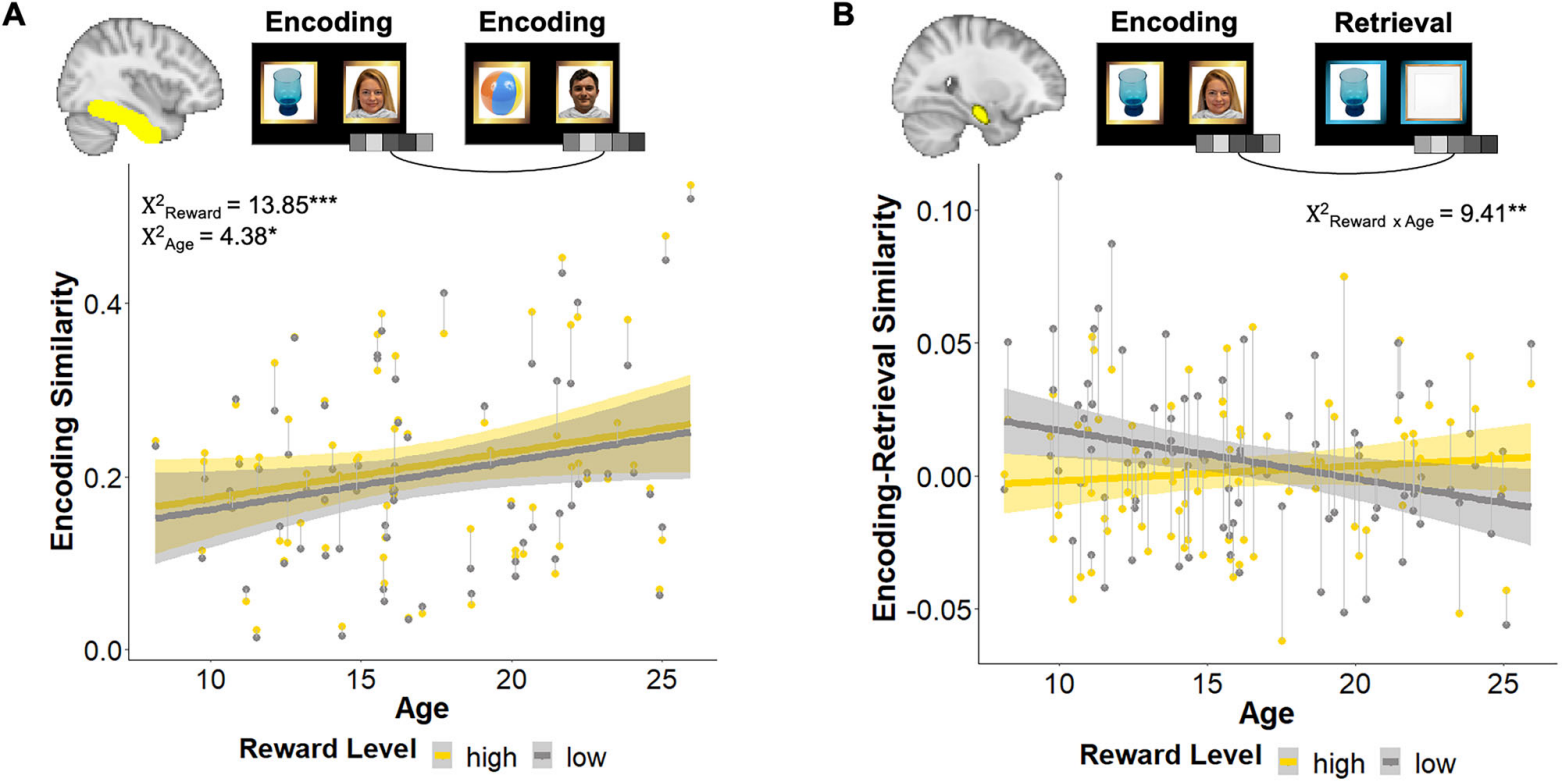
Reward-sensitive neural pattern similarity in VTC and aHC. ***A***, VTC encoding similarity increased with age and was increased for high- relative to low-reward memoranda across age. ***B***, aHC encoding-retrieval similarity varied by both reward level and age, such that pattern similarity for high-relative to low-reward memoranda was differentiated for younger and older participants (reward level by age interaction). Shading depicts 95% confidence intervals around fitted lines. Thin gray lines connect individual subjects’ data points. *p < 0.05; **p < 0.01; ***p < 0.001.

We first examined neural pattern similarity in VTC. Using a series of linear mixed-effect models, we examined VTC encoding similarity, retrieval similarity, and encoding-retrieval similarity as a function of the reward level and age, controlling for the high-reward source image category (faces vs places). We observed significant main effects of the reward level (*χ*^2^_(1, *N* = 73)_ = 13.85; *p* = 0.002) and age (*χ*^2^_(1, *N* = 73)_ = 4.38; *p* = 0.036) on encoding similarity, such that encoding similarity was greater for high- relative to low-reward memoranda and increased with age (Fig. 2*A*). There was no significant interaction between the reward level and age or an effect of high-reward source image category (*χ*^2^s < 1.2; *p*s > 0.23). We did not observe any significant differences in VTC retrieval similarity as a function of the reward level, age, or source image category (*χ*^2^s < 1.5; *p*s > 0.21). Finally, we only observed a significant main effect of quadratic age (*χ*^2^_(1, *N* = 89)_ = 7.02; *p* = 0.008) on VTC ERS such that similarity increased nonlinearly with age. There were no main effects of the reward level, linear age, high-reward source image category, or interactions between the reward level and age (*χ*^2^s < 1.6; *p*s > 0.20; see summary of findings in Table S2). These findings suggest that paired associates were encoded more similarly with a greater reward value and increasing age and that individual memory representations increase in similarity with age, regardless of the reward level.

### aHC encoding-retrieval similarity varies by the reward level and age

We next examined neural pattern similarity in our primary region of interest, the aHC. We used a series of linear mixed-effect models to assess whether aHC encoding similarity, retrieval similarity, and encoding-retrieval similarity varied as a function of the reward level and age, controlling for the high-reward source image category. There were no significant differences in either encoding or retrieval similarity in aHC as a function of the reward level, age, or source image category (all *χ*^2^s < 1.2; *p*s > 0.28). There were also no significant main effects of the reward level, age, or high-reward source image category on aHC encoding-retrieval similarity (*χ*^2^s < 1.6; *p*s > 0.22). However, there was a significant reward level by age interaction (*χ*^2^_(1, *N* = 89)_ = 9.41; *p* = 0.002), such that high-reward paired associates showed relatively greater encoding-retrieval similarity than low-reward pairs in older participants but less encoding-retrieval similarity relative to low-reward pairs in younger participants (Fig. 2*B*; see summary of findings in Table S2). To further examine this age-related difference, we conducted a follow-up analysis with age treated categorically—i.e., binned into children (8–12.99 years), adolescents (13–17.99 years), and adults (18–26 years)—and found this pattern held, with a significant interaction for reward by age group (adults vs adolescents, *β* = 0.017; *p* = 0.046; adults vs children, *β* = 0.029; *p* = 0.002). In a follow-up contrast examining the estimated marginal means, we found that children showed greater low-reward ERS than high-reward ERS (*p* = 0.008), adults showed a trend toward the opposite pattern (*p* = 0.094), and adolescents showed an intermediate pattern with no difference in ERS by the reward level (*p* = 0.28; Fig. S2). We also assessed whether aHC ERS patterns might differ by age, based on accuracy. A trial-wise mixed–effect model with memory accuracy, age, and reward level (and their interactions) predicting aHC ERS revealed no significant interactions of age by accuracy (*p* = 0.99), reward by accuracy (*p* = 0.97), or age by reward by accuracy (*p* = 0.83), suggesting the reward by age interaction on aHC ERS is not a function of difference in performance by age. Together, these findings suggest that adolescence may be a period of transition from child-like to more adult-like memory representations. These results suggest that individual memory representations in aHC were differentiated by the reward level in both older and younger individuals but with opposite patterns of neural similarity for stimuli from high- and low-reward categories as a function of age.

### VTC encoding-retrieval similarity predicts trial-wise memory accuracy

**Figure 3.**
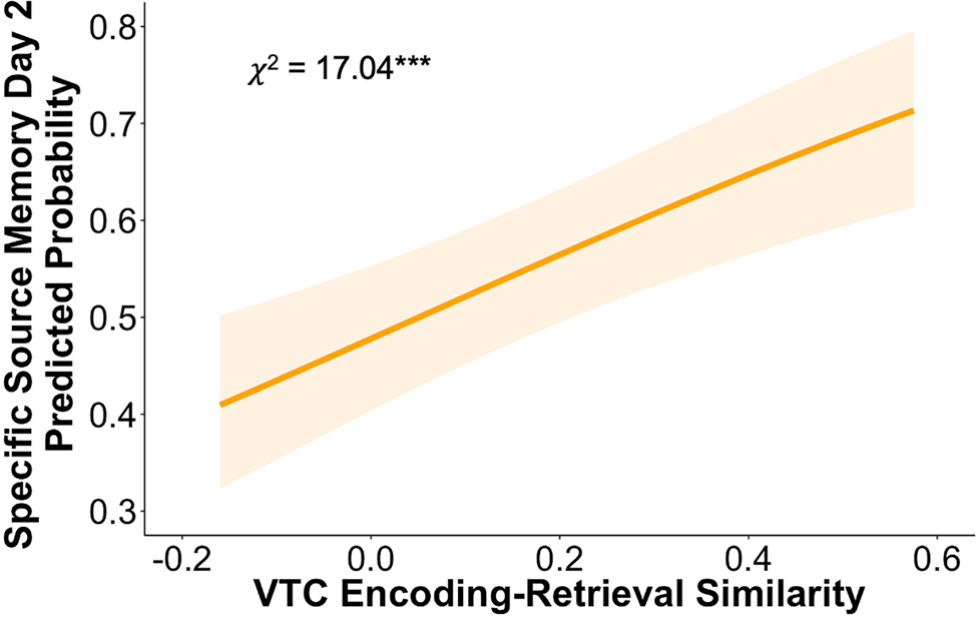
Trial-wise VTC pattern similarity relates to memory performance across age. Trial-wise VTC encoding-retrieval similarity was related to memory after 24 h, such that greater encoding-retrieval similarity predicted better memory performance. Shading depicts 95% confidence intervals around the predicted probability line. ***p < 0.001.

We next assessed whether VTC reinstatement was associated with trial-wise memory accuracy after 24 h. Specifically, we fit a generalized linear mixed-effect model to examine the trial-wise relation between VTC encoding-retrieval similarity and memory accuracy, controlling for the reward level, age, and high-reward source image category. As expected, we found a robust positive relationship between VTC encoding-retrieval similarity and memory performance (*χ*^2^_(1, *N* = 89)_ = 17.04; *p* < 0.001; Fig. 3), suggesting that the reinstatement of neural patterns from encoding in visual regions during retrieval may have supported successful memory for specific source images. As anticipated based on analyses of the behavioral data, we also found that memory performance increased with age (*χ*^2^_(1, *N* = 89)_ = 4.22; *p* = 0.04) and that high-reward associations were better remembered than low-reward associations (*χ*^2^_(1, *N* = 89)_ = 7.26; *p* = 0.007). There was no significant effect of source image category (*χ*^2^_(1, *N* = 89)_ = 0.01; *p* = 0.93). Thus, we find that VTC reinstatement supports memory performance regardless of age or the reward level of stimuli.

### Neural similarity measures relate to reward-motivated memory enhancements

We found that VTC encoding similarity and aHC encoding-retrieval similarity patterns both reflected an influence of reward value. To assess how each of these neural measures related to reward-motivated memory enhancements, we ran a multiple regression including the neural similarity measures from each of these two regions as covariates predicting memory performance after 24 h. Specifically, because we observed greater encoding similarity for high- versus low-reward items in VTC, which did not vary by age, we included the difference score between high- and low-reward encoding similarity in VTC as the covariate for this region. However, in aHC we observed reward value-related differentiation of encoding-retrieval pattern similarity that differed in older compared with younger participants, such that adults showed greater pattern similarity for high-reward pairs, while children showed lower similarity for these pairs. We reasoned that the magnitude of differentiation, regardless of the sign of the difference, might relate to the strength of the reward-modulated memory effect. Thus, for the aHC covariate, we computed the absolute value of the difference score between high- and low-reward encoding–retrieval similarity. The dependent variable in the model was a high-reward memory benefit measure, which was simply the difference between high- and low-reward memory performance after 24 h. Both the VTC and aHC difference scores were included as predictors, controlling for each other, along with age and the high-reward source image category.

**Figure 4.**
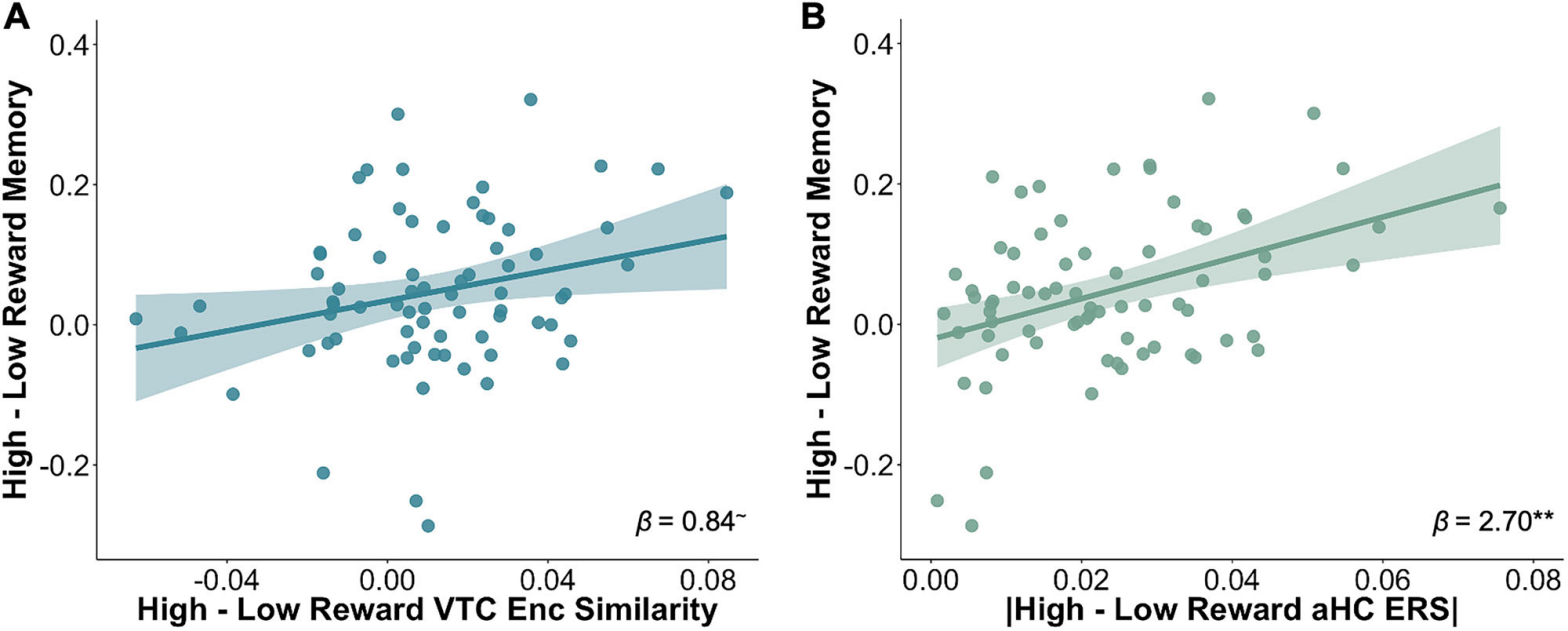
Reward-sensitive neural similarity measures relate to high-reward memory benefits. ***A***, Greater high- versus low-reward VTC encoding similarity was related to better high- versus low-reward memory after 24 h. ***B***, Greater magnitude of the difference between high- and low-reward aHC encoding-retrieval similarity was related to better high- versus low-reward memory after 24 h. Results come from a single multiple regression model. Shading depicts 95% confidence intervals around fitted lines. ∼*p* < 0.1; **p < 0.01.

There was a marginal positive relationship between high- and low-reward VTC encoding similarity and the high-reward memory benefit (*β* = 0.84; *t*_(68)_ = 1.82; *p* = 0.074; Fig. 4*A*), suggesting that the enhanced encoding similarity for high-reward items in VTC may support better memory for these items. We also observed a significant positive relationship between the magnitude of high- versus low-reward aHC encoding-retrieval similarity and the high-reward memory benefit (*β* = 2.70; *t*_(68)_ = 3.42; *p* = 0.001; Fig. 4*B*), suggesting that the magnitude of the difference between high- and low-reward ERS, regardless of sign, may facilitate memory for high-reward items. There were no significant effects of age or the high-reward stimulus category (*p*s > 0.80). These findings suggest that reward-sensitive adaptations of neural representations in both VTC and aHC support reward-motivated memory enhancements.

To assess whether the brain–behavior relationships were specific to the difference between high- and low-reward memory, we conducted two follow-up regression analyses, one examining high-reward memory as the dependent variable and the other examining low-reward memory as the dependent variable. We observed a significant effect of aHC ERS in the high-reward memory model (*p* < 0.001) but not the low-reward model (*p* = 0.932), indicating that aHC ERS effects are driven by better high-reward memory performance. We did not observe a significant effect of VTC encoding similarity in either model (*p*s > 0.22), indicating that the marginal effect of this neural measure in predicting memory was specific to the difference between high- and low-reward memory.

### VTA encoding activation relates to reward and age-varying hippocampal encoding-retrieval similarity

Because our prior work on this dataset (Cohen et al., 2022a) demonstrated that pre- to postencoding change in functional connectivity between aHC and VTA differentially predicted memory performance by age, we wanted to examine whether reward-related activation in VTA relates to aHC ERS differentially with increasing age. Given this increased putative communication between VTA and aHC following reward-motivated encoding, we reasoned that VTA high > low encoding activation may relate to the aHC representational pattern observed in younger participants more so than the pattern observed in older participants. While the magnitude of the difference between high- and low-reward aHC ERS robustly related to reward-motivated memory benefits across age, high-reward paired associates were represented more similarly in older participants, while low-reward paired associates were represented more similarly in younger participants (Fig. 2*B*). We examined whether univariate encoding activation of VTA for high- versus low-reward memoranda related to aHC ERS. Specifically, we used an a priori anatomically and functionally defined VTA ROI to examine activation in the high-reward > low-reward encoding contrast and related it to the difference in high- versus low-reward aHC ERS, with age and source image category as control variables. Here, we did not take the absolute value of the high–low hippocampal encoding-retrieval similarity metric specifically to attempt to capture potential relations between differential VTA activation and the more “child-like” or more “adult-like” pattern of differentiation of memory representations for pairs of stimuli from each reward level within the hippocampus.

**Figure 5.**
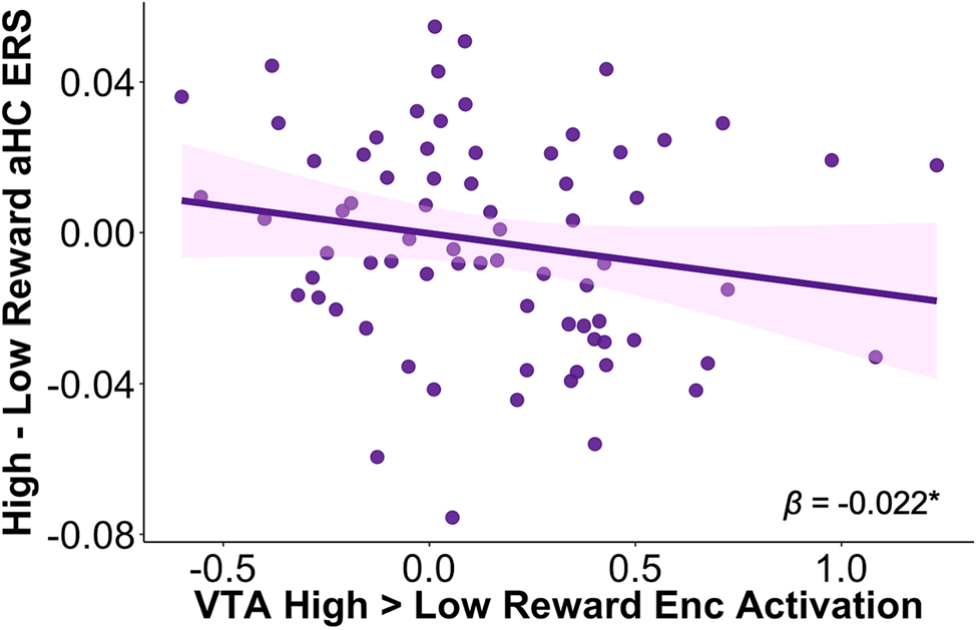
VTA encoding activation for high-reward memoranda relates to anterior hippocampal (aHC) high- versus low-reward pattern similarity. Greater VTA encoding activation for high- relative to low-reward memoranda was associated with lower encoding-retrieval similarity for high- relative to low-reward memoranda in aHC. *p < 0.05.

We found a significant relationship between VTA encoding activation and high- versus low-reward aHC encoding-retrieval similarity, such that greater VTA activation for high- versus low-reward stimuli was associated with increased low- relative to high-reward aHC encoding-retrieval similarity (*β* = −0.022; *t*_(68)_ = −2.27; *p* = 0.026; Fig. 5), which was the prevailing similarity pattern observed in younger participants (Fig. 2*B*). There was a marginal main effect of age (*β* = 0.007; *t*_(68)_ = 1.81; *p* = 0.075), such that high- versus low-reward aHC ERS increased with age. There was no significant main effect of the high-reward source image category (*p* = 0.75). In a follow-up analysis where we modeled the interaction of high > low VTA activation with age, there was no interaction (*p* = 0.54). Together, these findings suggest that greater VTA encoding activation for high-reward memoranda may facilitate representational drift of hippocampal reward-related neural patterns from encoding to retrieval and that although both reward-motivated aHC ERS and VTA activation increase with age (Fig. S3), the relationship between these two measures is not moderated by age.

## Discussion

In the present study, we investigated how reward influences neural memory representations across development. Specifically, we examined neural pattern similarity in the VTC and the aHC. We found that VTC encoding similarity and aHC encoding-retrieval similarity reflected reward value in different ways. VTC encoding similarity was greater for high- relative to low-reward memoranda and increased with age, regardless of the reward level. aHC encoding-retrieval similarity varied by the reward level as well as by age—mean neural pattern similarity between encoding and retrieval was higher for high-reward stimulus pairs and lower for low-reward pairs with age, such that older participants exhibited greater aHC similarity for high-reward memoranda whereas younger participants exhibited lower aHC similarity. The increase in VTC encoding similarity for high- relative to low-reward memoranda and the magnitude of the high- versus low-reward ERS difference in aHC were both associated with high-reward memory benefits after 24 h. Furthermore, greater high-reward encoding activation in VTA was associated with decreased similarity between neural patterns for high-reward memoranda from encoding to retrieval in aHC, the representational scheme that was more commonly observed in younger participants. These results highlight differences in how VTC, aHC, and VTA incorporate reward-related information within long-term memory and in how hippocampal representations support reward-motivated memory across development.

Although VTC encoding similarity predicted memory performance and related to reward value regardless of age, the degree of similarity for both high- and low- reward memoranda increased with age. One explanation for our findings comes from work demonstrating that VTC regions become more robust in their category-specific responses with increasing age, leading to the potential for greater similarity in response patterns for objects from the same reward category with development (Golarai et al., 2007; Grill-Spector et al., 2008). However, it has also been suggested that over development, multivariate neural representational similarity for items from the same category is present prior to the emergence of univariate neural category selectivity (Cohen et al., 2019), highlighting the provisional nature of this literature. Specificity in neural responses to objects, faces, and scenes in VTC has been demonstrated in infants, but category selectivity in these regions continues to develop into early adulthood (Grill-Spector et al., 2008). In particular, although object-selective neural activation and behavioral recognition are at adult-like levels by middle childhood, activation in face- and place-selective regions and memory for exemplars from these two categories continue to develop into adulthood (Golarai et al., 2007), which is proposed to be due to experience-related plasticity (Golarai et al., 2015; Nordt et al., 2021). Although category-selective functional activation grows stronger across childhood and adolescence, activation in VTC correlates with category-specific memory performance even among children as young as 7 years old (Golarai et al., 2007). Given that faces and scenes were the categories paired with each of the reward levels (low and high) in our study, increases in the degree of pattern similarity in VTC across development in our sample may reflect this protracted developmental trajectory of cortical representations.

Strikingly, reward facilitated memory in adults by promoting stability of neural patterns for high-reward stimuli, whereas children's memory was instead facilitated by lower similarity or “drift” in hippocampal activation patterns. Representational drift refers to shifts in neural patterns or “engrams” representing a particular stimulus over time (Rule et al., 2019), reflecting a distributed neural code that dynamically shifts with ongoing experience (Hainmueller and Bartos, 2018; Rule et al., 2019). A prior study reporting that reward expectation reduces drift of spatial representations in the CA1 subregion of HC in adult mice (Krishnan and Sheffield, 2023) is consistent with the greater hippocampal neural pattern similarity (i.e., reduced drift) for high-reward pairs we observed in adults. However computational accounts proposing that drift in the CA3 subregion of the HC may actually facilitate memory performance (Antony et al., 2024) suggest that stability does not always benefit memory, consistent with our finding that younger participants exhibited better memory but lower neural pattern similarity for high-reward pairs. The CA1 and CA3 subregions of the hippocampus exhibit distinct maturational trajectories (Lavenex and Banta Lavenex, 2013; Keresztes et al., 2018), and animal models indicate there may also be differences in the rates of representational drift across these regions (Hainmueller and Bartos, 2018). One possibility is that children's and adults’ comparable reward-motivated memory enhancements reflect differential contributions of dynamic representations within these HC subfields as the hippocampus continues to develop, a hypothesis that could be explored in future developmental studies using approaches that afford better spatial resolution of neural representations. Further work in this area may also clarify the nature of hippocampal representations in adolescence. We did not observe a prevailing reward-differentiating representational scheme employed by the hippocampus during adolescence. Adolescents’ value-related hippocampal memory representations may exhibit greater variation as teens transition from “child-like” to the “adult-like” patterns.

Themagnitude of reward-related VTA activity during encoding was related to the differential degree of stability versus drift in hippocampal patterns from encoding to retrieval for high- versus low-reward memoranda. Our prior work showed that pre- to postencoding increases in VTA–aHC functional connectivity were associated with reward-motivated memory to the greatest extent in younger participants (Cohen et al., 2022a), suggesting that increased communication between VTA and aHC following reward-motivated encoding was particularly beneficial for children's memory. Here, we found that greater VTA encoding activation for high-reward memoranda was associated with lower aHC pattern similarity for these stimuli from encoding to retrieval, the pattern that was observed to a greater extent in younger participants. However, in a direct test of the association between VTA encoding activation and aHC pattern similarity across age, we did not find this association to be stronger in younger participants relative to older participants. These results suggest that reward-related VTA activation during encoding may promote representational drift of high-reward memories in the hippocampus across participants of all ages. This finding highlights individual differences in the neural representations that support reward-motivated memory, consistent with findings in recent studies demonstrating individual variability in neural representations of pain (Kohoutová et al., 2022) and mindwandering (Kucyi et al., 2024).

The age-varying benefits of both representational drift and stability in aHC for reward memory raise numerous questions for future research. Emerging evidence from studies of associative memory supports the notion that distinct patterns of reinstatement and neural representation schemes could benefit motivated memory at different ages (Schlichting et al., 2022; Varga et al., 2025). Our findings also highlight substantial heterogeneity in hippocampal representational schemes, particularly across adolescence. Age-related and individual variability in memory representations may be due to differences in the cognitive representations reflected in anterior hippocampal activation patterns. For example, prior work indicates that, in adults, the hippocampus forms abstract, conceptual reward representations that benefit memory (Zeithamova et al., 2018). The extent to which memory is supported by conceptual versus perceptual reward representations may vary across development and across individuals. Furthermore, as outlined previously, it may be the case that representational schemes and content vary across hippocampal subfields. Future work that assesses both individual and developmental variabilities in reward memory representations is needed to test these hypotheses.

Reward associations impact the formation and retention of memory across the lifespan. Here, we provide evidence for developmental and individual differences in how reward influences the neural representations that support associative memory. Taken together, our findings align with a nascent body of research suggesting that distinct representational schemes benefit memory over development (Schlichting et al., 2022; Varga et al., 2025). Our work points to important avenues for future research into the mechanisms that underpin variation in the neural representations of motivated memories across age and between individuals.
